# A medium‐weight deep convolutional neural network‐based approach for onset epileptic seizures classification in EEG signals

**DOI:** 10.1002/brb3.2763

**Published:** 2022-10-05

**Authors:** Nazanin Nemati, Saeed Meshgini

**Affiliations:** ^1^ Department of Biomedical Engineering Faculty of Electrical and Computer Engineering University of Tabriz Tabriz Iran

**Keywords:** convolutional neural network, discrete wavelet decomposition, early seizure detection, EEG, medium‐weight structure

## Abstract

**Introduction:**

Epileptic condition can be detected in EEG data seconds before it occurs, according to evidence. To overcome the related long‐term mortality and morbidity from epileptic seizures, it is critical to make an initial diagnosis, uncover underlying causes, and avoid applicable risk factors. Progress in diagnosing onset epileptic seizures can ensure that seizures and destroyed damages are detectable at the time of manifestation. Previous seizure detection models had problems with the presence of multiple features, the lack of an appropriate signal descriptor, and the time‐consuming analysis, all of which led to uncertainty and different interpretations. Deep learning has recently made tremendous progress in categorizing and detecting epilepsy.

**Method:**

This work proposes an effective classification strategy in response to these issues. The discrete wavelet transform (DWT) is used to breakdown the EEG signal, and a deep convolutional neural network (DCNN) is used to diagnose epileptic seizures in the first phase. Using a medium‐weight DCNN (mw‐DCNN) architecture, we use a preprocess phase to improve the decision‐maker method. The proposed approach was tested on the CHEG‐MIT Scalp EEG database's collected EEG signals.

**Result:**

The results of the studies reveal that the mw‐DCNN algorithm produces proper classification results under various conditions. To solve the uncertainty challenge, K‐fold cross‐validation was used to assess the algorithm's repeatability at the test level, and the accuracies were evaluated in the range of 99%–100%.

**Conclusion:**

The suggested structure can assist medical specialistsin analyzing epileptic seizures' EEG signals more precisely.

## Graphical Abstract

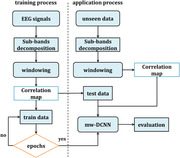

## 1 INTRODUCTION

Epilepsy is a painful disease that influences the nervous system, and subsequent seizures appear for the patient with the continuation (Capovilla et al., 2016). In some definition, seizures are known as sudden and transient abnormalities, leading to hallucinations, consciousness loss, and whole‐body seizure (de Lange et al., 2016). Mutation in a molecular mechanism is one of the reasons for the occurrence of onset of epileptic seizures, which conduce the brain damage malignant brain tumor, stroke, and infection. Clinical statistics indicate that epilepsy appears in 50 million people worldwide, and this neurological disorder is a crucial cause of mortality after Alzheimer's and stroke (Hill et al., 2015).

Human societies and families of patients with epilepsy pay exorbitant costs for care each year (Yu et al., 2019). These challenges justify the requirement for a novel approach to more conventional handle seizures that will serve both the person and their families responsible for the impacts and consequences of seizures. Proper and accurate diagnosis of this disease will help the patient, and on the other hand, the staggering costs of treatment and care of epileptic patients will be significantly reduced. The main reason why many automated methods are used in the early diagnosis of epilepsy is to find a way to predict the disease smoothly. The automatic identification of epileptic EEG signals is a helpful method for epileptic seizure diagnosis. Recent deep learning patterns not successful to fully examine diagnosis and disorder classification, which may lead to eliminating nonlinear and nonstationary characteristic in an epileptic. We need a therapeutic method, and thus, the therapeutic model should be capable of recognizing seizures at their onset stage. This model is grouped by the treatment used to slow the progression of seizures: local electrode stimulation (Li & Cook, 2018), thermal stimulation (Fernandes et al., 2018), or neurochemical stimulation (Wang et al., 2018).

Although intelligent approaches to evaluating epilepsy seizures have been widely proposed, deep learning techniques have been accepted in order to increase input signals and improve classification efficiency. The advantages of deep architectures are numerous: they do not require the signal or picture at the feature extraction level, making it possible to retain the input image and signal data. Additionally, development in response to the points indicated above serves the deep network architectures. Recently, Deep Neural Networks (DNNs) have been trained using appropriate feature extraction and transformation strategies to attain the necessary performance in classifying epilepsy occurrences (Rezaee et al., 2022; Rezaee et al., 2021). However, deep learning techniques are engaged in various fields, such as disease classification based on physiological signals, speech recognition, brain‐computer interface system (BCI), and other related items (Kiral‐Kornek et al., 2018; Nejedly et al., 2019). Accordingly, deep learning to study and analyze physiological signals is seen in many studies (Cho & Hyun‐Jong, 2020; Antoniades et al., 2016; Chowdhury et al., 2021).

Epilepsy is regarded as the most chronic, common, and severe neurologic disease, and therefore, some studies have utilized deep learning to recognize and process EEG signals. In Turner et al. (2014), the Deep Belief Networks (DBNs) were employed to distinguish the seizure events using recorded EEG signals from the multichannel analysis. Wulsin et al. (2011) also demonstrated that DBN structure could be used in a semisupervised classification procedure for modeling patterns and analyzing the EEG signals. Several researchers have proposed CNN designs to diagnose seizures using EEG signals (Johansen et al., 2016; Antoniades et al., 2016; Li et al., 2016; Amin & Kamboh, 2016). A robust deep learning technique based on stacked auto‐encoders (SAE) and the maximum entropy correlation function was presented for seizure detection (Qi et al., 2014). They tried to reduce noise and artifacts from sample EEG signals. The generalizability and efficiency of similar methods are not practicable and easily applied; nevertheless, the deep learning methods can be useful models to diagnose epileptic seizure onset.

Xiang et al. (2015) suggested using Fuzzy Entropy (FuzzyEn) as a method for studying epileptic seizures and diagnosing the disorder. In their work, classifying EEG data from patients with distinct epilepsy disorders needs first doing a Fuzzy Entropy analysis. In order to successfully train support vector machines with extracted features, a grid optimization technique was utilized in combination with a grid optimization method.

In Li et al. (2015), it was demonstrated that a method based on distribution entropy (DistEn) outperformed standard entropy approaches for detecting epileptic seizures via electroencephalogram (EEG) signals, particularly for short data lengths.

The EEG signals from normal and epileptic episodes were evaluated using an empirical mode decomposition (EMD) method (Pachori et al., 2015). The EMD generates internal mode functions, which are composed of a succession of modulated components.

According to Bhattacharyya and Pachori (2017), epileptic incidents can be diagnosed using multivariate oscillatory EEG data on adaptive frequency scales. The empirical wavelet transform (EWT) was applied to assess the amplitudes and frequencies of multivariate signals.

Raghu et al. (2019) claimed to have discovered a key feature of the EEG matrix determinant for detecting epileptic occurrences in patients. This study employed bivariate plots, polar coordinate histograms, and descriptive analysis.

Tzimourta et al. (2019) proposed using EEG data and the Discrete Wavelet Transform (DWT) to develop an automatic seizure detection approach. These coefficients can be used to denote the wavelet layers in each EEG segment.

Using Fourier–Bessel series expansion and weighted multiscale Renyi permutation entropy, Gupta and Pachori (2019) have developed a new approach to identify epileptic events by reconstructing rhythms from EEG data.

Sriraam et al. (2019) classified varied seizure forms into an eight‐class categorization scheme using CNNs. According to their findings, epileptic seizures can be classified as nonseizures, tonic‐clonic, tonic, absence, generalized nonspecific, focal nonspecific, difficult partial, and simple partial.

Sharma et al. (2020) developed a computationally efficient approach for determining the type of seizure. Consistent results were obtained using the proposed strategy. In their article, they used nonlinear higher‐order statistics and deep neural network algorithms to identify seizures.

de la O Serna et al. (2020) accomplished early diagnosis of epilepsy by utilizing rhythm‐specific EEG data as well as Taylor‐Fourier filter banks with O‐splines. The frequency response of the EEG signal was computed using Taylor‐Fourier subband signals, and the results are provided in their work.

Our main motivations and contributions are summarized in three parts: (1) DWT helps to optimize the classification performance by using frequency decomposition and variation detection of seizure and nonseizure pattern, (2) the mw‐DCNN are implemented as the EEG signal classification model to reduce the computational complexity, and (3) Design a generalizable model for classifying and diagnosing epilepsy using in‐depth learning that operates in the face of new signals with appropriate speed and satisfactory performance. Deep learning has eased epilepsy diagnosis and classification. Our study presents a useful classification approach. We use mw‐DCNN to improve decision‐making, and a discrete wavelet transform decomposes the EEG signal to detect the subbands of EEG signals. Moreover, CHEG‐MIT Scalp EEG signals are used to evaluate the proposed procedure. The mw‐DCNN algorithm has been tested in several circumstances. Besides, we assess the algorithm's repeatability at the test level, with accuracy between 99 and 100%. Among the contributions that this work is aiming to make are the following:

- The proposed new model significantly improves the framework's generalizability. Due to the system's robustness, epileptic incidents can be detected. By combining deep structure and decomposition methods, classification errors may be reduced.
- The method's computing cost has been lowered while preserving classification accuracy. Deep learning's structure minimizes computational complexity in decision making and enables it to work in real time or near real time.

The remainder of the article is organized in the following manner. The deep learning and decomposition process is discussed in Section 2. The overview and the proposed approach for detecting epileptic seizure are detailed in Sections 3 and 4. Finally, in Sections 5 and 6, the experimental results and conclusion are discussed.

## 2 DEEP LEARNING, DECOMPOSITION, AND SIGNAL ANALYSIS

Similar to Neural network (NN) designs, the ultimate output choice of a DNN, such as CNN, is based on the biases and weights of the prior layers in the network architecture (Rezaee et al., 2021). Therefore, the biases and weights of the deep architecture in CNN models are updated. In the convolution process, the feature map from the most recent layer is mixed with the kernel's feature map from the previous layer. Nonlinear downsampling techniques such as max pooling can be used to minimize the amount of feature maps generated by convolutional layers. When a feature map is imported into the max‐pooling layer, the max operation is applied to it, as illustrated in the figure. The feature map is used to update the maximum pooling layer. According to Equation ( (1), the procedure begins with the largest object.

(1) $$p_{j}=\underset{i\in Region_{j}}{\max\;\alpha_{i}},$$

in which *Region_j_* denotes the pooling region *j* contained within feature map *a* and *i* denotes the index of each contained element. Also, *p* denotes the pooled feature map. Multiclassification problems can also be solved using Softmax regression as (2):

(2) $$h_{{}_{\theta }}\left(x\right)={\left(1+e^{-\theta^{T}x}\right)}^{-1}.$$

The cost function is minimized by training the model parameters *θ*. Nonlinear processing layers are implemented in deep learning structures for feature extraction and transformation. The enabling hardware part (Elhosary et al., 2019), the architect of nonlinear designs (Birjandtalab et al., 2017), and model fine‐tuning procedures (Ullah et al., 2018) are between the growing viewpoints of the deep structure. The extracted pattern of seizure in EEG signals may differ from one patient to another.

The effects of extracted patterns from EEG signals in patients' seizure may be similar to the influence in nonseizure disease in other patients (Rezaee et al., 2016; Hassan et al., 2020; Dash et al., 2020). Decomposing the EEG data into different subbands and various frequencies will significantly lead to more valuable information due to the similarity in the patterns obtained from both types of signals. Hassan et al. (2020) proposed an automatic algorithm to diagnosis the epileptic seizure from EEG signals that, in the first step, they decomposed EEG signals into intrinsic mode functions. In their study, intrinsic mode functions were implemented by complete ensemble empirical mode decomposition with adaptive noise (CEEMDAN). The EEG signals are decomposed into gamma band with *f* > 30 Hz, beta band with 12 < *f* < 30 Hz, alpha band with 8 < *f* < 12 Hz, theta band with 4 < *f* < 8 Hz, and delta band with *f* < 4 Hz. Sharma et al. (2020) detect abnormal from seizure EEG signals by combining localized wavelet filter bank features and classification procedure.

**FIGURE 1:**
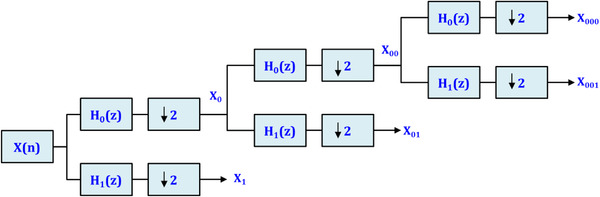
We have used discrete wavelet decomposition (DWT) method to decompose EEG signals into multiple subbands. The down arrow is downsampling by 2

They were yield reliability of 79.34% by finding the combination of extracted features from decomposed EEG signals. Jiang et al. (2020) also used decomposed techniques, including the symplectic geometric decomposition technique for epileptic EEG signal description. Raghu et al. (2019) utilized a computationally efficient automatic seizure diagnosis based on successive decomposition index (SDI) procedure. The decomposing EEG signals into subbands are different, and each can have its effects to obtain the proper features. However, as shown in Figure 1, we utilized the DWT to decompose sample signals.

## 3 MODEL OVERVIEW

**FIGURE 2:**
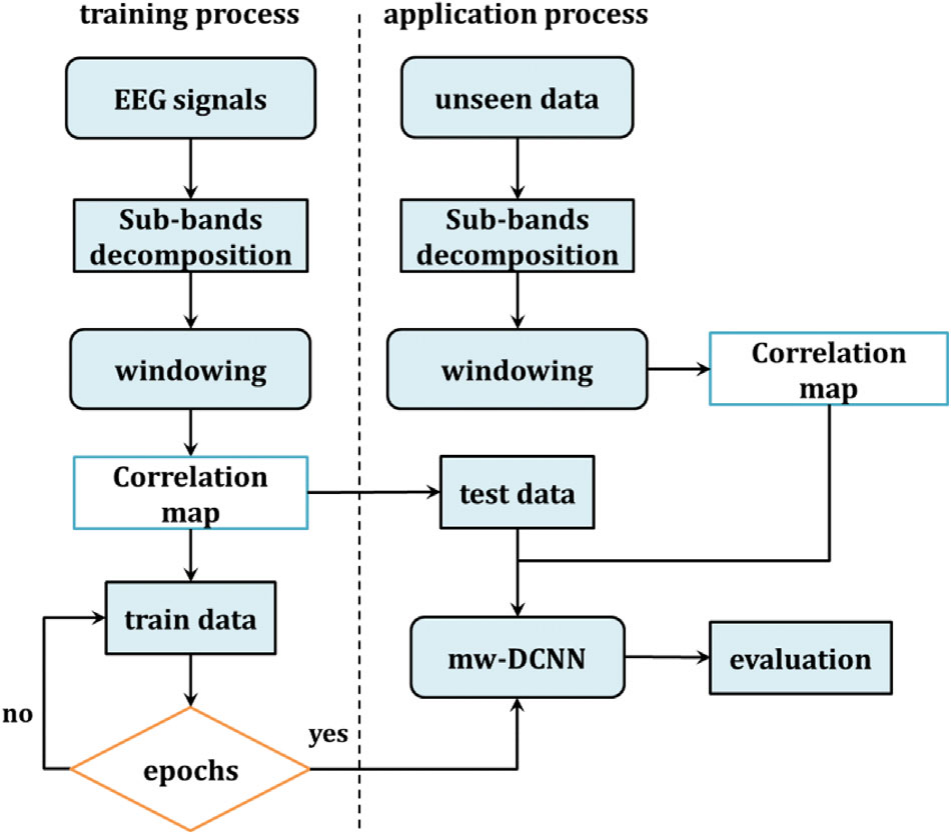
The schematic of introduced approach for identifying the onset epileptic seizures

The proposed model's overall structure is depicted in Figure 2. The DWT technique is employed to decompose sample signals to obtain various subbands to implement the proposed method.

The training procedure is determined by the input of signals and their subband decomposition, as shown in Figure 2. Signal windowing and correlation mapping are performed. The test phase is completed by considering the deep learning circumstances to acquire the appropriate response. The technique is examined using the test step, regardless of the feature maps constructed during the training phase.

Moreover, the DWT has a substantial advantage over other transforms, such as the Fourier transform, in that it extracts both time and frequency information from a signal simultaneously (Hadadnia & Rezaee, 2013; Subasi et al., 2019). The initial step in wavelet decomposition is to pass a time series signal through a range of high‐ and low‐pass filters. It is desirable to employ DWT because of its speed of processing and ease of implementation.

The deep learning structure is employed to classify patterns after analyzing the signal, improving the classification accuracy of epilepsy disorder. We show our algorithm based on separated steps, including training and test processes. The introduced model consists of wavelet decomposition and a robust framework for epileptic seizures classification. The deep learning structure is configured on a medium‐weight deep convolutional neural network (mw‐DCNN).

## 4 METHODOLOGY

**FIGURE 3:**
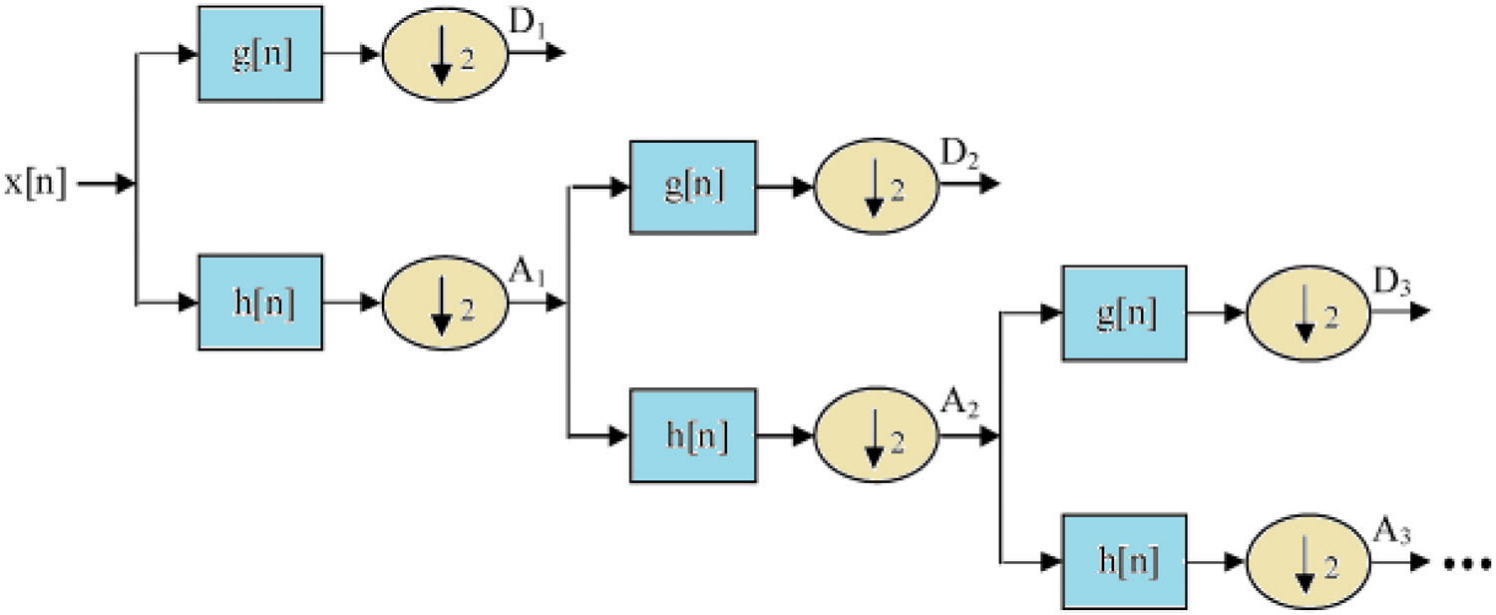
Utilization of *h*[*n*] and *g*[*n*] filters to decompose EEG signals (*x*[*n*]) into subbands

As presented in Figure 3, we convert the EEG signals into various subband data. In the next step, the subbands preprocessed by the DWT strategy. We use two filters and a downward sampler with a sampling coefficient of two rates. The *g*[.] is a primary inherently high‐pass wavelet and discrete method, and consequently, *h*[.] is defined as the mirror versions of the same wavelets. Also, the *h*[.] is an inherently low‐pass filter that is employed as a second filter in the decomposition procedure.

To segment and analyze the nonstationary EEG signals, an overlapped window technique is handled, which slides over the data with a predefined size and a preset increment *w*. In proposed design, we applied a window size of 300 ms with an increment of 20 ms. According to a study (Canolty et al., 2006), the EEG signal is time series correlated. As a result, the proposed approach initially feeds raw EEG data to the CNN. Additionally, we visualize the sample signal as a two‐dimensional array, with time steps denoting breadth and EEG electrodes denoting height. Additionally, some studies (Wei et al., 2018; Prathaban & Balasubramanian, 2021) employed the aforementioned technique to change the input's size.

### 4.1 The proposed learning

The suggested DCNN model is constructed of a hierarchical design that includes three layers of aggregate. The convoluted layer in first part is utilized to extract different solid features of the sample EEG signal. Besides, the additional two layers can develop higher surface characteristics, and individually feature mapping is formed of various inputs mapping with a convolution. The output can be defined as (3):

(3) $$x_{j}^{\ell }=f\left(\sum_{i\in M_{j}}x_{j}^{\ell -1}\times k_{ij}^{\ell }+b_{j}^{\ell }\right),$$

where *ℓ* depicts ℓ layer and *k_ij_* is the convolution kernel. Furthermore, *b_j_* shows the bias, and *M_j_* represents the collection of input mappings. Also, the sigmoidal function is illustrated in the mapping of the *j*th feature with the position (*x*, *y*) and the layer *i*th, *v_ij_^xy^* is described as (4):

(4) $$v_{ij}^{xy}=sig\left(b_{ij}+\sum_{p=0}^{P_{i}-1}\sum_{q=0}^{Q_{i}-1}w_{ij}^{xy}v_{\left(i-1\right)}^{\left(x+p)(y+q\right)}\right),$$

where *b_ij_* and *sig(.)* are the bias‐mapping functions and sigmoidal function, besides, *Q_j_* and *P_i_* are the width and height of the kernel, and finally, *w_ij_^pq^* is the kernel weight. The stochastic integration layer was employed. Reducing the variance and finding the maximum value of an appropriate feature in a domain of the EEG samples are the actual implementing appearance of the stochastic integration layer. Also, preventing the over‐fitting problem is another proper aspect of using mentioned layers.

**FIGURE 4:**
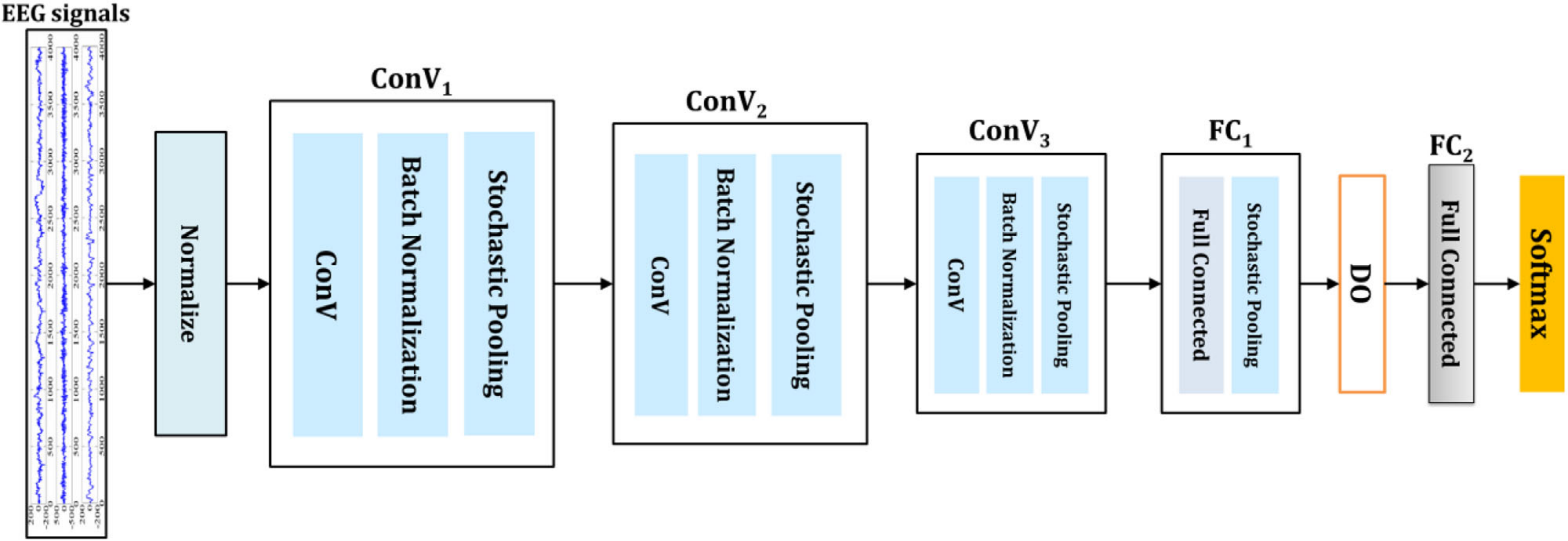
Proposed medium‐weight structure of the deep convolutional network

Following the layers of combination and convolution, a considerable number of inadequate feature maps are prepared. The network trained to analyze the signal status by importing all the training data and defining the label of epilepsy and nonepilepsy. Eventually, by joining these layers to the Softmax full connected layer, decision‐making is possible. These layers are considered as the input data, and thus, label is defined at the training step. At the training level, the system tries to determine the best‐unexplored parameters, involving filter weights and coefficients of layers. Therefore, the least error is reached in the classification step. The recursive descending gradient algorithm involves of two steps, including forward‐feeding (FF) and propagation error, and is also employed for training the network (Rezaee et al., 2020). First, we examine the difficulty to be of a two‐class representation, that the class *c* and *N* of the training signals are investigated. The squared error function (SEF) is further displayed by (5):

(5) Err=(1(122)∑n=1N∑k=1c(Tkn−Ykn)2,

where *T_kN_* and *Y_kN_* are the *k*th dimensions of *n*th design of the corresponding label and the predicted label returned by the CNN model, we utilize low number of layers. Notably, two layers are developed for adequate decomposition by DWT for EEG signals following the DCNN model. The proposed structure of the introduced network is shown in Figure 4. We implement multiple layers that include 4–12 layers. The first structure has 3–5 layers (lw‐DCNN), the second structure has 5–8 layers (mw‐DCNN), and finally, third structure has 8–12 layers (hw‐DCNN). The structure of CNN layers, the filter size, and the number of filters for CNN and max‐pooling operations are presented as layers 1–7. Convolutional layers (i.e., 1, 2, 4, and 6) are Conv1, Conv2, Conv3, and Conv4 with 10 × 1 (20 filters), 20 × 23 (20 filters), 10 × 20 (40 filters), and 10 × 40 (80 filters) respectively. Stochastic layers (i.e., 3 and 5) are 2 × 1 (stride 2) and 2 × 1 (stride 2), respectively. The decision layer is Softmax or dense layer with 2 and 3 classes.

### 4.2 Correlation map

**FIGURE 5:**
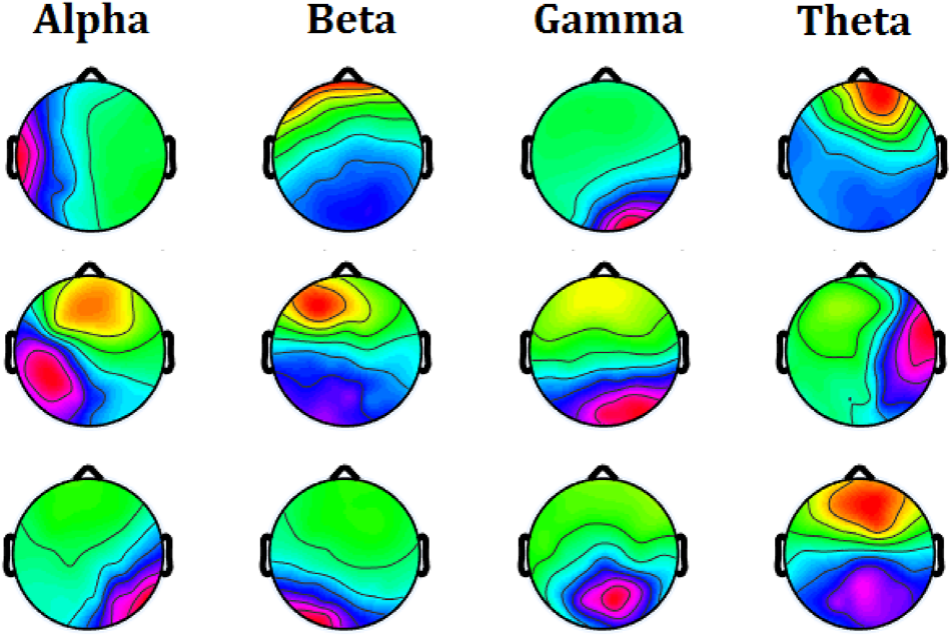
This figure depicts correlation maps of decomposed EEG signal for a patient

The major challenge of network learning is interpreting and understanding CNN performance. Consequently, it is challenging to determine what the structure is learning, how it is attaining such prominent outcomes, and what kinds of features utilize for classification. Thus, we analyze the model to visualize and computations for CNN. The subbands of EEG signals contribute the discriminative information for the classification of motor imaginary signals. Figure 5 depicts correlation maps of decomposed EEG signal for a patient.

The amplitudes of frequency subbands are employed to compute the medium values for different frequencies and engaged as feature quantities. Accordingly, the mean values of features are related within a receptive area for each layer of the Convolutional Network as the usual spectral amplitude with the initial output for each layer. Thereupon, the calculated correlation maps are a measurement of the collection of the spectral amplitude of a unit. The calculated correlation maps are analyzed to the layer output to figure out which of the features are applied by CNN. By changing the information as artificial data, the features and amplitude are also modified, and we can understand whether there is a variation in the output of the CNN.

## 5 EXPERIMENTAL RESULTS

The introduced strategy was performed on a workstation with Intel (R), Core (TM) and Core i7 processors with a 64‐bit operating machine and 4 GB of RAM. We utilized MATLAB programming software (2019b version) and presented the results in the form of quantitative and qualitative outputs.

The near frequency spans of decomposed signal to multiple subbands consist of 0–4 Hz, 4–7 Hz, 8–13 Hz, and 13 to 30 Hz ranges (Liu et al., 2012). The EEG signal parts were converted the 5 levels of decomposition into approximations and details coefficients to produce a more effective discriminative model among the nonseizure and seizure signals in the various frequency subbands. The range of seizure signals was extensive and happened in the frequency span of [3 Hz–29 Hz] after the decomposition (Peker et al., 2015). The layers H1 through H4 are made up of 200 neurons with 104 weights, 300 neurons with 12 weights, 400 neurons with 112 weights, and lastly 192 neurons with 24 weights. The network's activation function was also chosen as the hyperbolic tangent activator. The convolution layers, which are multidimensional tensors, are converted to a unidimensional tensor using Soft‐extended Max's fused full connected output layer. Finally, weights are improved via RMSprop optimization, which is a technique for determining a variety of optimization functions.

### 5.1 Data set

The CHB‐MIT database was generated solely by Children's Hospital Boston (CHB) for this study (Goldberger et al., 2000). Twenty‐four children with uncontrollable epileptic convulsions were recorded using electroencephalography (EEG). Twenty‐three EEG instances, ranging in age from 1.5 years to 22 years old, are included in this collection of 916 h of EEG recordings. This collection consists of recordings and cases ranging in age from 1.5 years to 22 years. This collection of recordings has been separated into three distinct parts for the sake of organization and accessibility. For youngsters as young as 1.5 years old, there are recordings in the collection. There are people in the database as young as 1‐month‐old and as old as 22 years old. One month is how long the database is on average. In order to see if the seizures were disappeared, an EEG scan was performed. This and the discovery of what they had done shocked them. There are about 600 recordings in CHB‐EEG MIT's database, making it a substantial collection. One hundred ninety‐eight people have been documented in these files, including information on two different types of seizures. The most common sort of seizure is a nonseizure, while seizures are quite rare. For a maximum of 4 h, this collection's data is stored on a disk. At 256 samples per second, the 16‐bit precision EEG was used to record each patient's EEG. The International 10−20 method, developed by UK academics, was used to record EEG signals on the scalp. A lab can be set up to record and analyze EEG waves from the scalp. Physionet, a medical research website, has this information (Goldberger et al., 2000).

### 5.2 Assessments

We used accuracy, sensitivity, and specificity criteria to evaluate the epilepsy seizure detection model according to Equations (6)–(8):

(6) $$\begin{array}{ccc} & & Accuracy=\\ & & \left(\frac{N_{TruePositive(TP)}+N_{TrueNegative(TN)}}{N_{TruePositive(TP)}+N_{TrueNegative(TN)}+N_{FalseNegative(FN)}+N_{FalsePopsitive(FP)}}\right), \end{array}$$

(7) $$Sensitivity=\left(\frac{N_{TruePositive(TP)}}{N_{TruePositive(TP)}+N_{FalseNegative(FN)}}\right),$$

(8) $$Specificity=\left(\frac{N_{TrueNegative(TN)}}{N_{TrueNegative(TN)}+N_{FalsePositive(FP)}}\right).$$

Tables 1, 2, 3, 4 have been depicted the outcomes of the classification scheme for five times trials of the design in two categories for the theta, gamma, beta, and alpha subbands, respectively. In mentioned tables, the results show the effect of the decomposition strategy on the input signal. The approximate standard deviation and accuracies have also been evaluated in low, medium, and high numbers of high layers based on the used weights of each structure. In general, the initial signal decomposition increases the classification performance in all cases. In other words, the design of the DCNN occurs with the best accuracy. If the length of each window on the signal is assumed to be 300 ms, 165 windows will be obtained for a 30‐s signal with 40% overlap between windows. Since the sampling frequency is 256 Hz, each window will have 75 step times for feature extraction. This means that we have 165 × 200 windows for each subject, and since there were 24 subjects in the test, the 3 classes consisted of about 790,000 windows.

**TABLE 1:** Evaluations of accuracy under a range of data division scenarios, both with and without the DWT technique, as well as changes in the number of deep layers in the alpha subband

Data dividing	No. layers	Without decomposition			With decomposition		
		**Best**	**Mean**	**Worst**	**Best**	**Mean**	**Worst**
10‐fold (1)	High	0.89 ± (0.03)	0.87 ± (0.04)	0.85 ± (0.04)	0.95 ± (0.01)	0.93 ± (0.04)	0.92 ± (0.04)
	Med	0.86 ± (0.03)	0.85 ± (0.05)	0.83 ± (0.05)	**0.96 ± (0.02)**	0.93 ± (0.03)	0.92 ± (0.04)
	Low	0.87 ± (0.03)	0.86 ± (0.03)	0.84 ± (0.03)	0.95 ± (0.01)	0.93 ± (0.03)	0.93 ± (0.04)
10‐fold (2)	High	0.89 ± (0.03)	0.85 ± (0.05)	0.83 ± (0.04)	0.96 ± (0.01)	0.94 ± (0.03)	0.92 ± (0.04)
	Med	0.88 ± (0.03)	0.84 ± (0.03)	0.83 ± (0.03)	**0.97 ± (0.01)**	0.94 ± (0.01)	0.92 ± (0.03)
	Low	0.87 ± (0.04)	0.85 ± (0.04)	0.84 ± (0.03)	0.96 ± (0.02)	0.93 ± (0.01)	0.91 ± (0.03)
10‐fold (3)	High	0.86 ± (0.03)	0.85 ± (0.04)	0.83 ± (0.03)	0.95 ± (0.01)	0.94 ± (0.01)	0.91 ± (0.02)
	Med	0.86 ± (0.03)	0.84 ± (0.03)	0.83 ± (0.05)	**0.96 ± (0.01)**	0.94 ± (0.04)	0.93 ± (0.04)
	Low	0.90 ± (0.04)	0.86 ± (0.05)	0.84 ± (0.06)	0.95 ± (0.02)	0.94 ± (0.03)	0.93 ± (0.04)
10‐fold (4)	High	0.88 ± (0.02)	0.86 ± (0.03)	0.85 ± (0.05)	0.95 ± (0.02)	0.94 ± (0.03)	0.92 ± (0.03)
	Med	0.86 ± (0.03)	0.85 ± (0.03)	0.84 ± (0.03)	**0.96 ± (0.01)**	0.93 ± (0.04)	0.92 ± (0.05)
	Low	0.90 ± (0.04)	0.87 ± (0.05)	0.86 ± (0.04)	0.95 ± (0.01)	0.94 ± (0.04)	0.90 ± (0.05)
10‐fold (5)	High	0.89 ± (0.03)	0.87 ± (0.03)	0.86 ± (0.04)	**0.97 ± (0.02)**	0.95 ± (0.04)	0.90 ± (0.04)
	Med	0.91 ± (0.03)	0.88 ± (0.03)	0.87 ± (0.04)	0.96 ± (0.01)	0.95 ± (0.03)	0.93 ± (0.03)
	Low	0.88 ± (0.04)	0.86 ± (0.05)	0.84 ± (0.06)	0.96 ± (0.03)	0.95 ± (0.03)	0.92 ± (0.03)

*Note*: The values in bold are the most precise.

*Note*: A bolded value represents the best value obtained.

**TABLE 2:** Evaluations of efficiency in various data dividing conditions and with and without DWT strategy and variation in the number of deep layers for beta subband

Data dividing	No. layers	Without decomposition			With decomposition		
		**Best**	**Mean**	**Worst**	**Best**	**Mean**	**Worst**
10‐fold (1)	High	0.91 ± (0.04)	0.90 ± (0.05)	0.88 ± (0.05)	**0.96 ± (0** **.01)**	0.94 ± (0.05)	0.93 ± (0.06)
	Med	0.91 ± (0.03)	0.90 ± (0.06)	0.88 ± (0.06)	0.95 ± (0.02)	0.94 ± (0.05)	0.93 ± (0.06)
	Low	0.93 ± (0.04)	0.90 ± (0.05)	0.87 ± (0.06)	0.95 ± (0.02)	0.93 ± (0.04)	0.92 ± (0.05)
10‐fold (2)	High	0.92 ± (0.04)	0.90 ± (0.06)	0.87 ± (0.05)	0.97 ± (0.03)	0.93 ± (0.04)	0.92 ± (0.05)
	Med	0.89 ± (0.04)	0.88 ± (0.05)	0.87 ± (0.05)	**0.97 ± (0.02)**	0.94 ± (0.05)	0.93 ± (0.04)
	Low	0.88 ± (0.03)	0.87 ± (0.03)	0.86 ± (0.05)	0.96 ± (0.02)	0.95 ± (0.04)	0.94 ± (0.04)
10‐fold (3)	High	0.87 ± (0.04)	0.86 ± (0.05)	0.85 ± (0.05)	0.96 ± (0.03)	0.93 ± (0.04)	0.92 ± (0.04)
	Med	0.85 ± (0.03)	0.84 ± (0.05)	0.83 ± (0.06)	**0.96 ± (0.02)**	0.94 ± (0.03)	0.93 ± (0.03)
	Low	0.88 ± (0.03)	0.87 ± (0.04)	0.85 ± (0.06)	0.95 ± (0.02)	0.94 ± (0.04)	0.92 ± (0.05)
10‐fold (4)	High	0.90 ± (0.03)	0.88 ± (0.05)	0.86 ± (0.06)	**0.97 ± (0.03)**	0.95 ± (0.04)	0.94 ± (0.04)
	Med	0.92 ± (0.03)	0.89 ± (0.04)	0.86 ± (0.06)	0.96 ± (0.02)	0.94 ± (0.05)	0.92 ± (0.05)
	Low	0.91 ± (0.05)	0.89 ± (0.04)	0.87 ± (0.05)	0.96 ± (0.03)	0.93 ± (0.05)	0.92 ± (0.04)
10‐fold (5)	High	0.90 ± (0.02)	0.89 ± (0.05)	0.87 ± (0.06)	0.96 ± (0.02)	0.94 ± (0.05)	0.93 ± (0.05)
	Med	0.90 ± (0.02)	0.89 ± (0.04)	0.88 ± (0.07)	**0.97 ± (0.03)**	0.94 ± (0.04)	0.92 ± (0.04)
	Low	0.89 ± (0.02)	0.87 ± (0.05)	0.86 ± (0.06)	0.95 ± (0.02)	0.96 ± (0.04)	0.93 ± (0.05)

*Note*: A bolded value represents the best value obtained.

**TABLE 3:** Evaluations of efficiency in various data dividing conditions and with and without DWT strategy and variation in the number of deep layers for gamma subband

Data dividing	No. layers	Without decomposition			With decomposition		
		**Best**	**Mean**	**Worst**	**Best**	**Mean**	**Worst**
10‐fold (1)	High	0.93 ± (0.04)	0.92 ± (0.05)	0.89 ± (0.07)	0.98 ± (0.01)	0.97 ± (0.03)	0.94 ± (0.04)
	Med	0.93 ± (0.03)	0.92 ± (0.05)	0.90 ± (0.06)	**0.99 ± (0.01)**	0.97 ± (0.03)	0.94 ± (0.04)
	Low	0.94 ± (0.03)	0.93 ± (0.06)	0.89 ± (0.07)	0.98 ± (0.02)	0.96 ± (0.03)	0.95 ± (0.04)
10‐fold (2)	High	0.92 ± (0.04)	0.92 ± (0.06)	0.89 ± (0.06)	0.98 ± (0.01)	0.96 ± (0.02)	0.94 ± (0.04)
	Med	0.94 ± (0.03)	0.92 ± (0.05)	0.89 ± (0.06)	**0.99 ± (0.01)**	0.96 ± (0.02)	0.93 ± (0.03)
	Low	0.93 ± (0.04)	0.92 ± (0.05)	0.90 ± (0.06)	0.97 ± (0.02)	0.96 ± (0.03)	0.95 ± (0.04)
10‐fold (3)	High	0.92 ± (0.03)	0.90 ± (0.06)	0.89 ± (0.06)	0.97 ± (0.02)	0.95 ± (0.03)	0.94 ± (0.03)
	Med	0.94 ± (0.04)	0.92 ± (0.05)	0.89 ± (0.05)	**0.98 ± (0.01)**	0.96 ± (0.04)	0.93 ± (0.04)
	Low	0.92 ± (0.05)	0.92 ± (0.06)	0.87 ± (0.05)	0.98 ± (0.02)	0.95 ± (0.03)	0.93 ± (0.06)
10‐fold (4)	High	0.93 ± (0.04)	0.91 ± (0.06)	0.89 ± (0.05)	0.99 ± (0.01)	0.97 ± (0.03)	0.94 ± (0.03)
	Med	0.94 ± (0.04)	0.92 ± (0.05)	0.90 ± (0.06)	**0.99 ± (0.01)**	0.97 ± (0.04)	0.95 ± (0.04)
	Low	0.94 ± (0.04)	0.91 ± (0.05)	0.90 ± (0.06)	0.99 ± (0.01)	0.97 ± (0.03)	0.93 ± (0.05)
10‐fold (5)	High	0.92 ± (0.03)	0.91 ± (0.06)	0.89 ± (0.05)	**0.99 ± (0.01)**	0.97 ± (0.03)	0.93 ± (0.04)
	Med	0.93 ± (0.04)	0.90 ± (0.06)	0.88 ± (0.06)	0.98 ± (0.01)	0.95 ± (0.04)	0.92 ± (0.05)
	Low	0.93 ± (0.05)	0.90 ± (0.05)	0.88 ± (0.07)	0.98 ± (0.01)	0.97 ± (0.02)	0.93 ± (0.04)

*Note*: A bolded value represents the best value obtained.

**TABLE 4:** Evaluations of efficiency in various data dividing conditions and with and without DWT strategy and variation in the number of deep layers for theta subband

Data dividing	No. layers	Without subband decomposition			With subband decomposition by DWT		
		**Best**	**Mean**	**Worst**	**Best**	**Mean**	**Worst**
10‐fold (1)	High	0.92 ± (0.03)	0.91 ± (0.04)	0.89 ± (0.05)	0.97 ± (0.02)	0.95 ± (0.04)	0.94 ± (0.05)
	Med	0.93 ± (0.03)	0.91 ± (0.04)	0.90 ± (0.06)	**0.98 ± (0.02)**	0.96 ± (0.04)	0.95 ± (0.04)
	Low	0.92 ± (0.04)	0.91 ± (0.05)	0.89 ± (0.07)	0.96 ± (0.03)	0.95 ± (0.02)	0.93 ± (0.04)
10‐fold (2)	High	0.91 ± (0.04)	0.90 ± (0.06)	0.88 ± (0.07)	0.97 ± (0.02)	0.95 ± (0.04)	0.94 ± (0.05)
	Med	0.91 ± (0.03)	0.90 ± (0.06)	0.88 ± (0.07)	**0.97 ± (0.01)**	0.95 ± (0.03)	0.92 ± (0.04)
	Low	0.92 ± (0.03)	0.89 ± (0.04)	0.87 ± (0.07)	0.96 ± (0.02)	0.94 ± (0.02)	0.93 ± (0.04)
10‐fold (3)	High	0.92 ± (0.04)	0.91 ± (0.05)	0.88 ± (0.06)	0.96 ± (0.02)	0.94 ± (0.04)	0.93 ± (0.05)
	Med	0.91 ± (0.03)	0.89 ± (0.05)	0.88 ± (0.06)	**0.98 ± (0.02)**	0.96 ± (0.02)	0.94 ± (0.05)
	Low	0.93 ± (0.02)	0.89 ± (0.06)	0.87 ± (0.06)	0.97 ± (0.03)	0.96 ± (0.04)	0.95 ± (0.04)
10‐fold (4)	High	0.91 ± (0.03)	0.90 ± (0.05)	0.88 ± (0.05)	0.97 ± (0.02)	0.95 ± (0.02)	0.93 ± (0.04)
	Med	0.91 ± (0.03)	0.89 ± (0.06)	0.87 ± (0.06)	**0.98 ± (0.01)**	0.96 ± (0.04)	0.94 ± (0.03)
	Low	0.92 ± (0.03)	0.90 ± (0.04)	0.87 ± (0.07)	0.98 ± (0.02)	0.97 ± (0.04)	0.95 ± (0.04)
10‐fold (5)	High	0.91 ± (0.04)	0.89 ± (0.05)	0.87 ± (0.06)	0.97 ± (0.02)	0.96 ± (0.05)	0.95 ± (0.05)
	Med	0.93 ± (0.03)	0.91 ± (0.05)	0.88 ± (0.07)	**0.98 ± (0.02)**	0.95 ± (0.03)	0.94 ± (0.05)
	Low	0.94 ± (0.03)	0.91 ± (0.04)	0.89 ± (0.06)	0.97 ± (0.02)	0.96 ± (0.04)	0.94 ± (0.03)

*Note*: A bolded value represents the best value obtained.

Higher frequencies are usually more commonplace in abnormal conditions for epilepsy in which there is a position alteration of EEG signal energy from lower to higher frequency subbands before and throughout a seizure occurrence. Following wavelet decomposition of the spectrum EEG signal, the extracted features from each subband independently. Hence, onset epilepsy seizures from nonstationary signals are easier to distinguish, mainly due to higher amplitudes. The selection of a proper wavelet and the number of decomposition stages is also extremely momentous in any analysis of EEG signals utilizing the wavelet transform. We computed the wavelet coefficients for all five various subbands of EEG signals. The tabulated confusion matrix (CM) beyond all 10‐folds (CV = 10) is displayed in Tables 1, 2, 3, 4. In these tables, it is perceived that 98% of the three classes of EEG signals are precisely classified as onset epilepsy seizure.

The classification proposed structure classified normal, onset epilepsy seizure, and certain seizure EEG data sets with an accuracy of 97%, 98%, and 99%, respectively. Overall, the EEG signals have been classified with an accuracy of 99%, which is the final classification accuracy by mw‐DCNN in different subbands. The eventuated classification accuracy of the proposed mw‐DCNN is quite high and therefore has the potential for a real clinical application.

**FIGURE 6:**
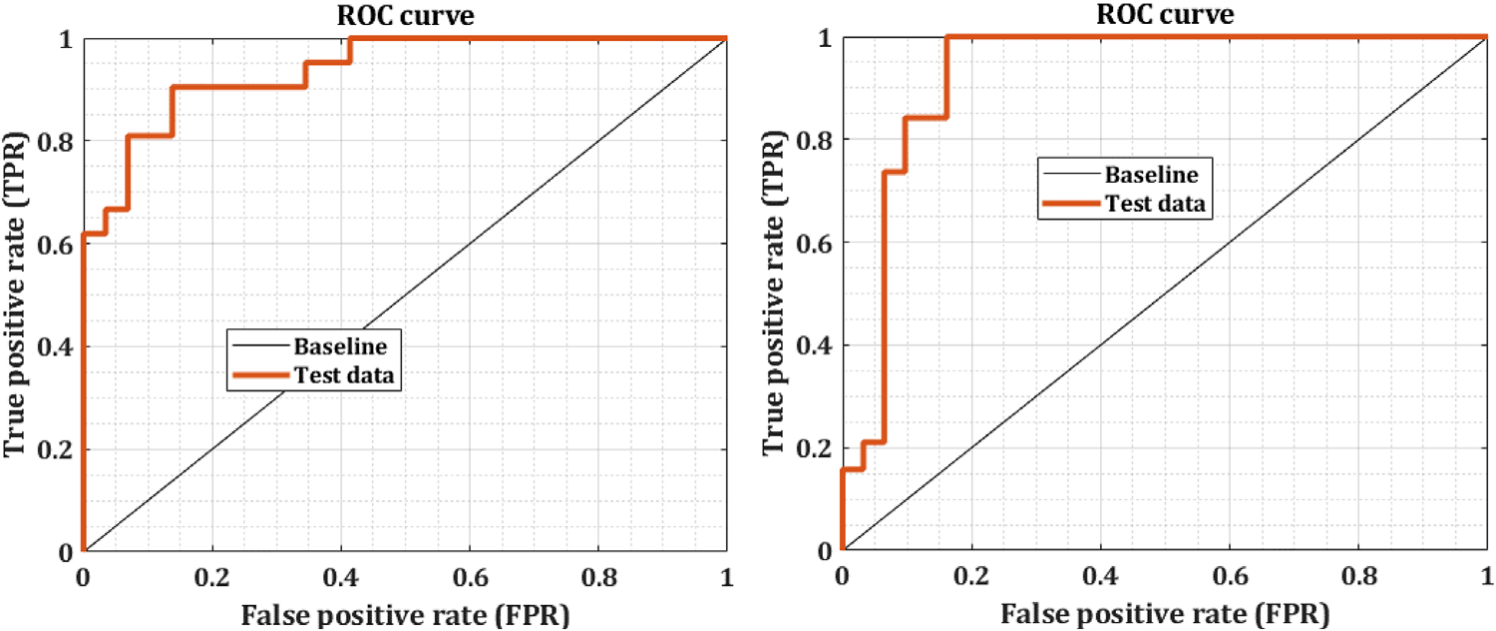
The ROC curve of two test EEG signals

**FIGURE 7:**
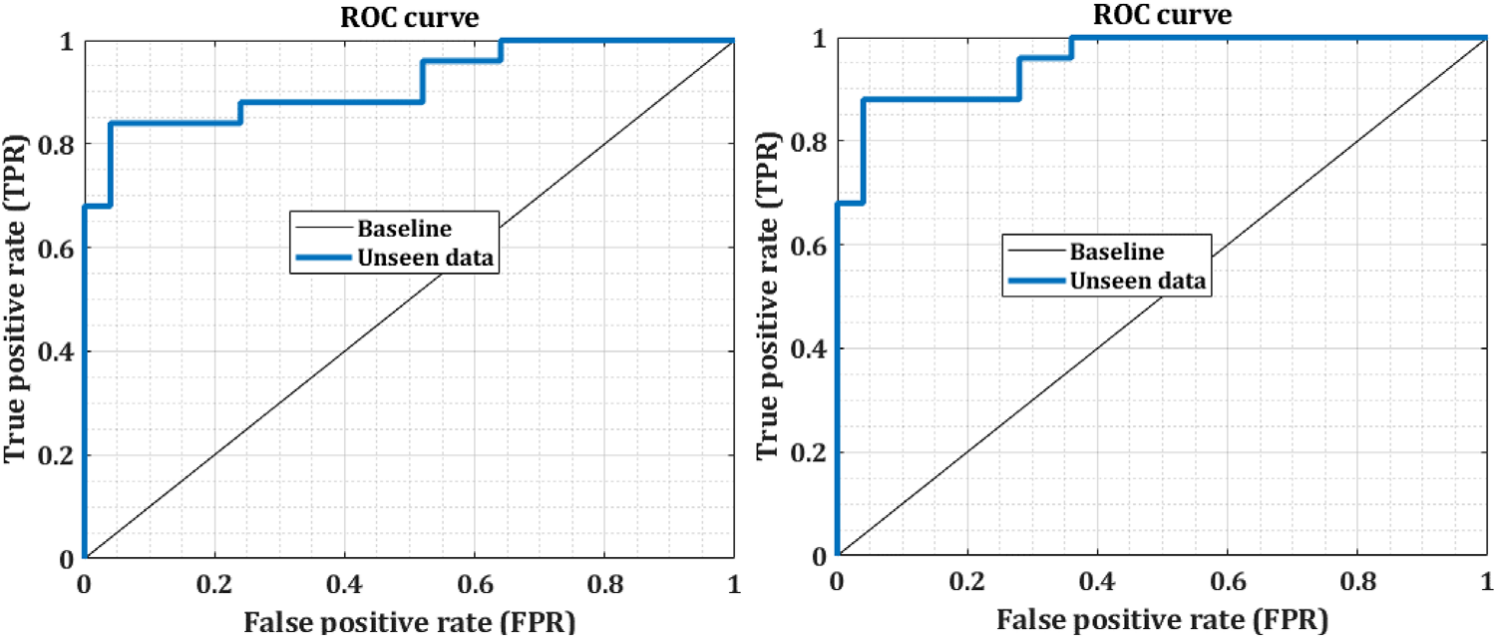
The ROC curve of two unseen EEG signals

The receiver operating characteristic (ROC) curve is engaged to assess the accuracy of a continuous measurement for predicting a binary outcome. The basic aim of visually illustrating the ROC curve is to demonstrate the trade‐off between the FPF and TPF as the cutoff *c* varies. We investigate two classes (i.e., nonseizure and onset epilepsy seizure) in our study to indicate ROC curve for test and unseen EEG signals. There are several summary assessments of accuracy and sensitivity associated with the ROC curve, namely the partial area under the curve at a fixed TPR and FPR, respectively (see Figures 6 and 7 for illustration). These figures show the mean ROC curves received using 10‐fold cross‐validation for various durations when an mw‐DCNN classifier with a medium layer was utilized. To compute and quantify the various runs, we plotted the AUC. Statistical significance tests using paired *t*‐tests demonstrate that the AUCs acquired applying low, medium, and a high number of layers are not statistically diverse from the AUCs acquired using similar deep learning methods.

## 6 DISCUSSION

**FIGURE 8:**
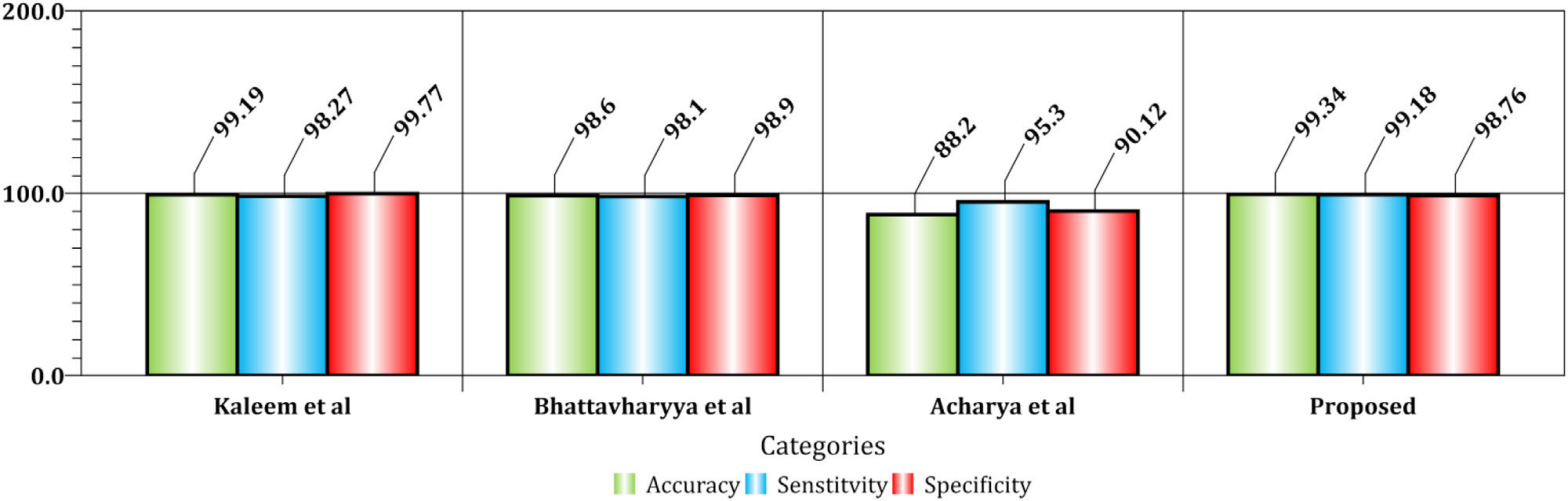
Similar methods were compared to the recommended strategy in order to determine the onset of an epileptic episode

Compared to similar methods, the proposed algorithm asserts that it can be effective for seizure analysis in EEG signal analysis with lesser expert knowledge. The investigation further demonstrated that mv‐DCNN could be a potent pattern to identify the onset of epilepsy seizure based on EEG signals. The proposed model attains state‐of‐the‐art efficiency on seizure patient detectors, learning a generic description of onset epilepsy; therefore, the proposed model provides meaningfully enhanced cross‐patient detection outcomes. We further observe in Figure 8 a qualitative comparison among the related systems. The suggested approach is associated with three comparable designs. The designs consist of the patterns of Kaleem et al. (2018), Bhattacharyya et al. (2017), and Acharya et al. (2018) that each of these techniques from the single SVM classifier, the adjustable Q wavelet, and deep learning is composed of several numbers of the layer in DNN structure, respectively. Instead of two categories classification, the three categories classification, including multiple severity of the seizures, was performed as another experiment.

**FIGURE 9:**
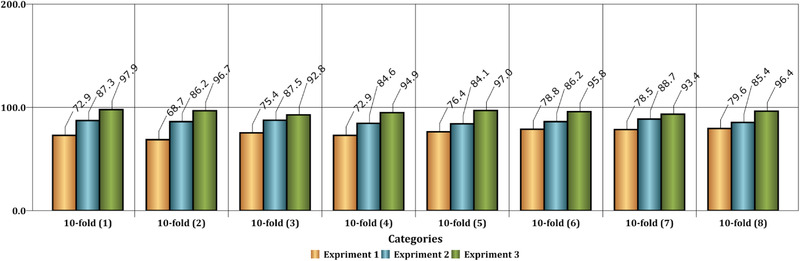
This figure shows the comparison of the accuracy of the classification with three classes. Experiment 1 has been performed without decomposition of input signals. Experiment 2 has been carried out with decomposition by DWT on input signals, and also, the network has a large number of layers. Finally, Experiment 3 has been conducted with decomposition by DWT on input signals, and also, the network structure has a low number of layers

The results are shown in Figure 9 by focusing on recognizing the onset of seizures as a multiclass problem. In this figure, various situations such as nonoptimal classification, optimal condition, and analysis based on decomposed signals are considered.

**FIGURE 10:**
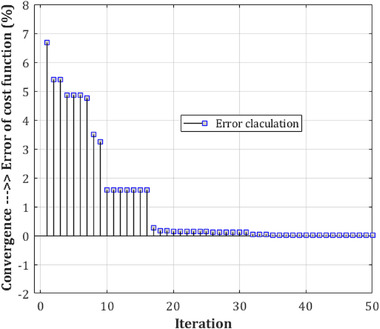
mw‐DCNN convergence in reducing the error in training data—random data 1 after 31 iterations and reaching the minimum value of zero in the classifier cost function

**FIGURE 11:**
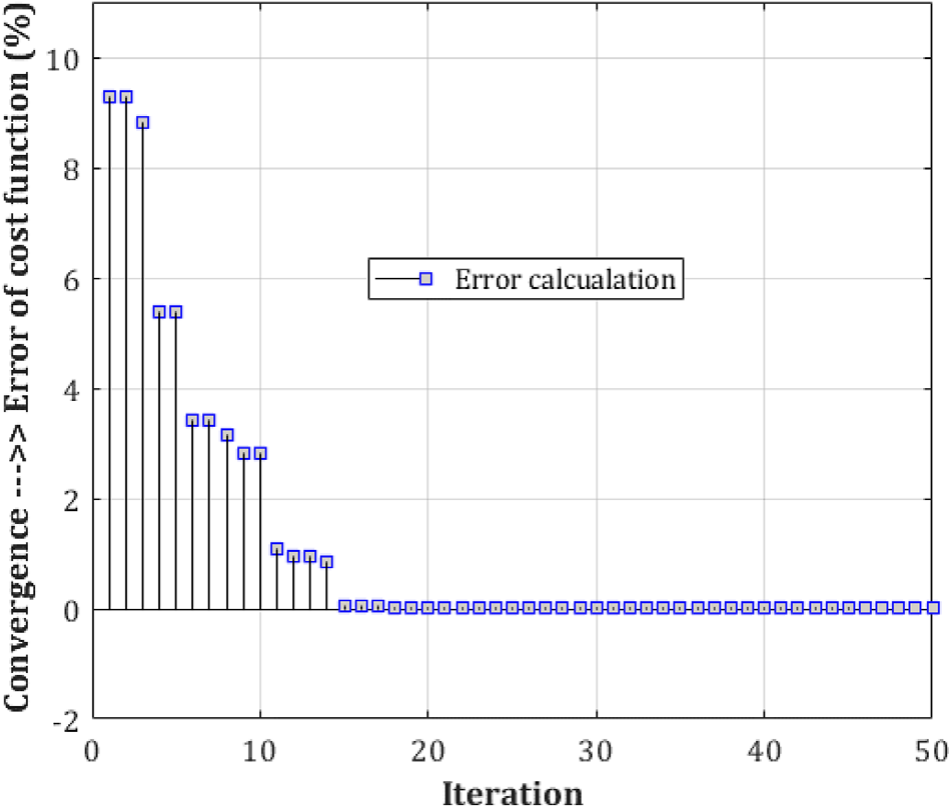
mw‐DCNN convergence in reducing the error in training data—random data 2 after 14 iterations and reaching the minimum value of zero in the classifier cost function

**FIGURE 12:**
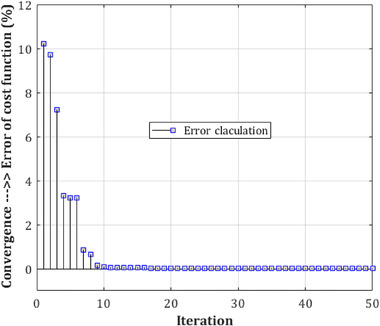
mw‐DCNN convergence in reducing the error in training data—random data 3 after 8 iterations and reaching the minimum value of zero in the classifier cost function

A comparison of similar methods reveals that the proposed procedure is extremely reliable. Nonetheless, when compared to the system of Acharya et al. (2018), the classification performance appears to have significantly improved. Because of the aforementioned circumstance, the method may have a larger positive error rate (PER) than their advised technique, but the supplied method's negative error rate (NER) is determined to be lower. Furthermore, the difficulty of trusting the projected labels was established. In recursive circumstances, the difference between the two test results was minimal. This signifies the events' suitability and dependability. Examination of variance and standard deviation for the two‐class classification category was carried out twenty times with K‐fold test, with values of 1.44 × 10^−4^ and 0.0153. In each iteration, various conditions of the number of layers were considered, where the medium number of deep CNN structure layers demonstrate the time spent to process in the lowest state during the training stage and, consequently, during the test level. Hence, the converged level of the error is evaluated based on the cost of the loss function (i.e., the loss function is proportional to the estimation of the classification error) of CNN for the limited number of epochs in Figures 10, 11, 12 for the complexity resulting from a low number of layers in the training step.

The accuracy obtained by the proposed mw‐DCNN design is higher than the current approaches for EEG onset epilepsy seizure classification. The specificity and sensitivity criteria are additionally more useful than similar approaches.

In Table 5, we compare the provided model to state‐of‐the‐art approaches for EEG seizure diagnosis and onset epilepsy classification. Additionally, Shoeb (1981) provides a patient‐specific approach for epilepsy onset that yields a 96% accuracy rate on identical EEG readings. The use of recurrent and CNN for cross‐patient detection of initial epilepsy seizures was described in Thodoroff et al. (2016), with a sensitivity of 85%. Similarly, Wilson et al. (2004) described a method for cross‐patient onset epilepsy episode categorization that relies on fuzzy neural networks (FNN) and meets the sensitivity criteria of 75%.

**TABLE 5:** The comparison of similar approaches with the introduced model on the CHB‐MIT database

Study	Method	Accuracy	Sensitivity	Onset epilepsy seizure
Thodoroff et al. (2016)	CNN+RNN	95%	85%	No
Hossain et al. (2019)	Deep CNN	98.05%	90%	No
Shoeb (1981)	SVM classifier	96%	–	Yes
Kaleem et al. (2018)	DWT decomposition—five classifiers	99.6%	99.8%	No
Bhattacharyya et al. (2017)	TQWT decomposition—kNN and SVM classifiers	99%	99%	No
Acharya et al. (2018)	CNN (13 layers)	88.67%	95.00%	No
Wilson et al. (2004)	Fuzzy‐NN	–	76%	No
Fergus et al. (2016)	k‐NN classifier	93%	88%	No
Supratak et al. (2014)	Stacked auto‐encoder	High false positive	100%	No
Xun et al. (2015)	SVM	88.8%	–	No
Proposed	DWT decomposition—mwDCNN	99.34%	99.18%	Yes

Fergus et al. (2016) proposed *k*‐NN classifier for seizure classification and achieved 93% accuracy and 88% sensitivity criteria. The obtained results in our CNN model are better than similar approaches in the classification of seizure disorder. Although in our model, the features and effect of them investigated as correlation maps to visualize the feature learning, the visualization of outputs was processed to measure correlation in Chowdhury et al. (2021). When applied to cross‐patient EEG data, the Chowdhury et al.’s (2021) technique yields a 98.05% overall accuracy, a 91.65% specificity, and a 90.00% sensitivity. Besides, Chowdhury et al. (2021) present a 99.46% accurate strategy for diagnosing seizures. However, it took around 5 h to complete 90 epochs of training and testing on each patient's cross‐patient signals. In contrast to their method, the brain mapping process identifies only significant and relevant features for seizure onset classification.

Some studies (Kaleem et al., 2018; Bhattacharyya et al., 2017; Acharya et al., 2018; Supratak et al., 2014; Stober, 2017) proposed a way to understand the features and weights learned by the CNN model. In other words, they strived to discover which EEG signals have the most efficacies on the convolution maps. As a result, our method aids in visualizing the specific orientation of band power features following decomposition of EEG signals. Furthermore, we can apply correlation maps as the input of the deep learning technique to classify onset epilepsy seizure and similar EEG signals. It should be noted that windowing and decomposition of EEG signals have facilitated the classification procedure of the nonstationary EEG signals in onset epilepsy seizure detection. Our method also obtained satisfactory specificity and sensitivity criteria, which means that the process algorithm has generalized well. Moreover, combining various machine learning approaches and optimization algorithms appears to improve classification performance (Tavasoli et al., 2021). However, combining deep learning algorithms can significantly improve the classification of various epilepsy signals (Abdelhameed & Bayoumi, 2021).

The fundamental disadvantage of DWT is that it analyzes signals using a predetermined function, which limits its adaptability. Another concern is that laboratory‐based real‐time EEG recordings comprise both brain activity and noise signals. Additionally, EEG seizure patterns vary significantly between patients and even within the same patient over time.

One of the major benefits of this study is that it might be utilized in hospitals or clinics to automatically detect epileptic EEG patterns. This capacity aids in the selection of antiepileptic medications as well as the determination of prognosis. The proposed technique, on the other hand, lowers human error and computing complexity while maintaining excellent classification accuracy.

## 7 CONCLUSION

We introduced a generic structure for EEG onset epilepsy seizure EEG signals analysis and classification applying medium‐weight deep CNN. The introduced procedure is a system based on a DCNN technique with a medium‐weight model and initial decomposition of input signals by the DWT method. The labels predicted by the proposed method are significantly correlated to the opinions of the neurologist, and thus, by applying unseen data, we overcame challenges such as uncertainty. The accuracy of identifying epileptic seizures for the current study for a two‐class problem, including the presence or absence of disease, was estimated to be greater than 99%. Besides, the accuracy in different recurrences was estimated to be more than 98% on average. This method, which uses deep learning with an average number of layers, requires fewer signals for training, and on the other hand, can be used as a robust system in clinical research and early detection of epileptic seizures.

The authors intend to continue developing the system in the future, focusing on real‐time design and noise resistance. The authors will refine and incorporate the existing technique for announcing the initial seizure notice on a medical diagnosis platform into future research. In the future, the method may aid neurologists in detecting and treating the underlying neurological problem shown in the disease's EEG signal more successfully.

## DECLARATION

None.

## CONFLICT OF INTEREST

We have declared that we do not have any conflicts of interest.

### PEER REVIEW

The peer review history for this article is available at https://publons.com/publon/10.1002/brb3.2763.

## Data Availability

The codes and data are all available from the corresponding authors.
